# A Legal and Forensic Medicine Approach to Police Physical Intervention Techniques in High-Risk Situations

**DOI:** 10.3390/ijerph17082809

**Published:** 2020-04-19

**Authors:** José C. Vera-Jiménez, José A. Lorente, Lucas González-Herrera, José A. Álvarez, Marta Ferreiro-González, Jesús Ayuso

**Affiliations:** 1 Municipal Police of Cadiz, Police Technology Area, Public Safety School of Council of Cadiz, 11010 Cadiz, Spain; 2 Department of Legal Medicine, Toxicology and Physical Anthropology; University of Granada, 18016 Granada, Spain; jose.lorente@genyo.es (J.A.L.); lgh@ugr.es (L.G.-H.); 3 Department of Physical Chemistry, Faculty of Sciences, INBIO, University of Cadiz, 11510 Puerto Real, Spain; joseangel.alvarez@uca.es (J.A.Á.); jesus.ayuso@uca.es (J.A.); 4 Department of Analytical Chemistry, Faculty of Sciences, IVAGRO, University of Cadiz, 11510 Puerto Real, Spain; marta.ferreiro@uca.es

**Keywords:** police force, operational tactical procedures, physical threats, arrest and self-defense skills, prevention of leaf injuries, legal and forensic medicine

## Abstract

*Background*: The physical intervention techniques (PITs) typically used by the police in troublesome situations are examined in terms of injuring potential depending on whether they target a body zone of high, medium or low vulnerability. Based on legal and forensic considerations, and principles of congruence, opportunity and proportionality, a need exists to favor opponent locking and arrest techniques targeting non-vulnerable zones to minimize the risk of severe damage. *Methods*: A search of the training manuals for the different kind of law of enforcement officers was carried out. Revision of injuries was available from electronic databases of academic o medical journals. *Results*: Three different locking and arrest PITs based on operational tactical procedures (OTP) that avoid zones of high or medium vulnerability are proposed. The new techniques use blocking, diverting and grabbing of the upper and lower limbs, followed by dislocation and locking of the same targets. *Conclusions*: The damaging potential of such PITs was assessed in terms of anatomical region and most were found to have a high risk of severe damage. The alternative PITs proposed here, which rely on OTP, improve in legal and forensic medical terms on existing choices and dramatically reduce the risk of injuring arrestees.

## 1. Introduction

Arresting a person in a troublesome situation inevitably requires some physical contact between officer and opponent that may be difficult to control in terms of applied strength. In fact, the typical physical intervention techniques (PITs) often requires striking or pressing and may thus result in injury depending on the targeted body zone and on the strength with which it is stricken or pressed. From a legal and forensic perspective, the human body contains a number of points of highly vulnerability hitting or pressing of which is highly likely to result in irreversible damage or even death. These areas of high vulnerability include injuries resulting from head or brain trauma [1,2,3,4,5], injuries of spine [6,7,8,9,10], neck, throat and airway [11,12,13,14,15,16,17,18,19], problems of blood pressure [20,21] or cardiopulmonary [22,23,24,25,26,27], injuries of rib [28], abdominal [29,30]. Fatal damage [31,32,33,34,35,36,37] and injuries resulting from fighting sport practicing [38] are also included in this high vulnerability area.

**Figure 1:**
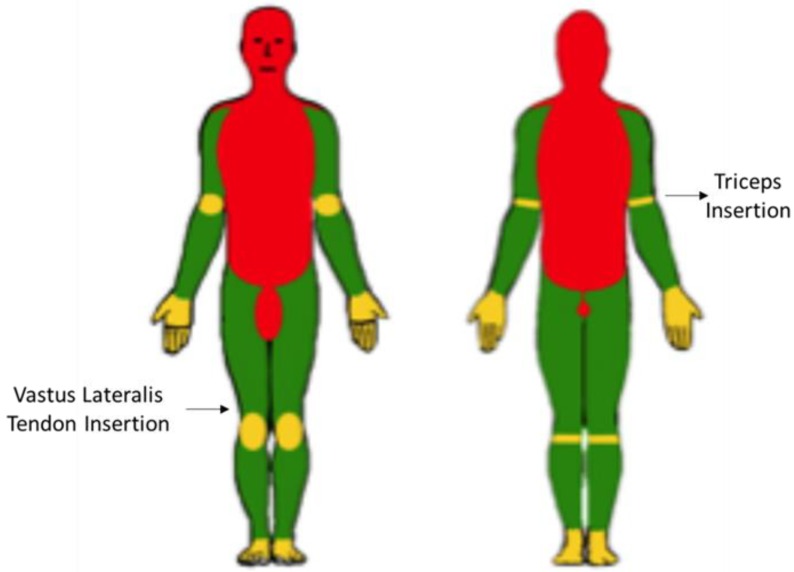
Zones of the human body hitting, handling or pressing of which can lead to variably serious damage. Red zone: points of high vulnerability (severe injury or death). Yellow zone: points of medium vulnerability (serious or permanent damage). Green zone: points of low vulnerability (no serious injury or permanent damage to be expected) [44].

The body also has points of medium vulnerability [39,40] in soft regions which should only be targeted if acting on points of low vulnerability [41,42,43] (viz., those where little or no injury is to be expected) is impossible. These three types of points fall in the red, yellow and green zone, respectively, of Figure 1 which was made based on the consulted references.

Highly vulnerable points are located in five different body regions, namely: head, spine, chest, abdomen and genitals. The head, neck, precordial zone—which houses the heart—abdomen and genitals are vital zones inasmuch as their hitting or heavy pressing may lead to highly severe injuries.

Hitting the head can obviously injure an individual because it contains so delicate an organ as the encephalon. Thus, trauma of the cranial dome or the face, with or without fracturing, can lead not only to external injuries in the scalp or facial skeleton and concussion, but also to hemorrhage and/or very serious encephalic damage that may result in immediate or delayed death, or in severe nerve damage. Striking the head should therefore be avoided at any rate [1,2,3,4,5].

The spine, which is the central pillar of the human body, encompasses a cervical, a dorsal and a lumbar portion. Because it contains the spinal cord (a vital component of the central nervous system that governs all body functions), this bony structure is highly prone to trauma. Spinal trauma can range from mild injury of vertebrae or vertebral muscles (fracture, crushing) to severe cord injury (total or partial splitting) leading to paralysis (paraplegia or quadriplegia depending on the extent of the damage). The cervical region is the most vulnerable of the three spinal components, not only because damage in it may result in quadriplegia but also because the neck itself is a vital zone containing vascular structures (the carotid arteries and jugular veins) whose compression or damage may lead to poor brain blood flow and loss of consciousness in addition to severe hemorrhage [6,7,8,9]. The neck additionally contains major nerve structures whose damage may result in serious nerve disorders. This is especially the case with the carotid sinuses, which fall at the carotid artery bifurcation under the mandibular angle. Thus, the sinuses contain baroreceptors triggering of which in response to, for example, a blow, can generate an impulse reaching vasomotor and vagal brain sites—and parasympathetic triggering of such sites can decrease heart rate (bradycardia) and inhibit a sympathetic response, thereby leading to vasodilation and a drop in blood pressure. In fact, striking a sinus can result in fainting through a decrease in heart rate and blood pressure or even lead to death through cardiac arrest [10,11,12,13,20,33,34,35,36]. Finally, the cervical zone comprises the upper airway, larynx and trachea, pressing or damage of which may result in severe complications such as tracheal or hyoid bone fracture and, ultimately, death through suffocation. Officers should therefore avoid striking their opponent’s neck in order to avoid a potentially fatal injury [14,15,16,17,18,19].

The chest not only comprises bony structures such as the sternum and ribs, but also houses vital organs such as the heart and lungs. Chest trauma often takes the form of rib fractures, which are usually very painful but rarely severe unless a number of bones are broken. By contrast, lung trauma can be very serious even in the absence of injury because it can cause contusions and/or lung hemorrhage, pneumothorax or haemothorax, all of which may be very serious or even fatal [21,22]. Similarly, heart trauma can result not only in contusion and direct injury of the organ, but also in rate disturbances such as commotion cordis, a serious event of arrhythmia (ventricular fibrillation) that may arise from a straight blow in the chest (specifically, in the precordial zone) and usually leads to death within minutes [23,24,37]. Officers should therefore also avoid striking the chest in order to preserve their opponents’ lives and safety [25,26,27,28].

Hitting the abdomen can inflict damage to various viscera especially serious among which is that resulting from spleen or liver fracture—a surgical, life-threatening emergency owing to the resulting massive hemorrhage [29,30]. The abdomen additionally contains major nerve structures such as the solar plexus, which is a vast network of nerves surrounding the aorta at the first lumbar rib behind the stomach. The epigastrium is a powerful reflex-triggering zone by effect of the solar plexus housing sympathetic and parasympathetic nerve fibers—so much so that epigastrial trauma can trigger responses decreasing heart rate and blood pressure to seriously low levels or even causing death through cardiorespiratory arrest [31,32,38].

The genitals are not only highly sensitive, but can also trigger an inhibitory vagal reflex, and it is well known that such an effect can lead to cardiac arrest [20], so they constitute another vulnerable zone for hitting.

In addition to the previous vulnerable zones and points, hitting of which should be avoided at all costs, the body contains zones of medium vulnerability that are located largely in the upper and lower limbs (hands, wrists, elbows, knees and feet) and fall in the yellow zones of Figure 1. Striking these zones can easily lead to injury but the resulting damage is rarely severe. Because most of the joints in the limbs are highly prone to injury, they should be acted upon with special care [39,40].

Finally, so-called zones of low vulnerability (the green zones in Figure 1) are also located in the upper and lower limbs but consists mostly of large muscles (e.g., femoral quadriceps, glutei, brachial triceps) whose hitting can cause acute pain but no serious injury. Other scarcely vulnerable zones include the anterior tibial edge and large joints such as the shoulders, which are less readily injured by locking. Trauma in a “green” zone can obviously cause injury (e.g., contusion, ecchymosis, rupturing of fibrils, tendons or muscles); the outcome, however, is rarely as serious as that of striking vulnerable or vital zones [41,42,43].

In this study, three different locking and arrest PITs based on operational tactical procedures (OTP) which avoid zones of high or medium vulnerability are proposed as alternative physical intervention techniques to avoid serious injuries or even death while ensuring that an opponent is arrested without damage to the police officer.

## 2. Materials and Methods

### 2.1. Training Manuals

For this study, a select bibliography from the forensic field has been consulted regarding the types of injuries resulting from sports activities [1,2,3], or from brain trauma [4,5], and of spine [6,7,8,9,10], of neck, throat and airway [11,12,13,14,15,16,17,18,19], of blood pressure [20,21], cardiopulmonary [22,23,24,25,26,27], of rib [28], abdominal [29,30], as well as fatal damage such as carotid sinus syndrome [31,32,33,34,35,36,37], injuries from fighting sport practicing [38,39,40,41,42], and also related to the prevention of damage in sport [43] and PITs [44,45]. A collection of training manuals for different types of security forces and bodies has also been compiled from various countries [46,47,48,49,50,51,52,53,54]. This type of study is appropriate to investigate the relationship between damage and injury to citizens, and the teaching of current PIT techniques.

### 2.2. Simulation of High-Risk Situations

For testing the proposed physical intervention techniques, two police officers were required (one who acted as a police officer and the other one as an opponent). Three different troublesome situations that police have commonly to face were recreated:

- A hooking punch. In this first scenario, the opponent stood in front of the police and tried to punch him in the face.
- A knife blow. The second scenario recreated the typical situation where a criminal is holding a sharp knife. After the police officer asked him to throw the knife away and calm down, the criminal who is cornered, tries to stab the knife on the police abdomen.
- A blunt object blow. In this last situation the criminal tried to hit the policeman with a blunt object blow like a baseball bat.

In each of the three situations the police officer reacted to applying the proposed operational tactical procedures (OTPs) in order to practice self-defense. The physical intervention techniques were displayed in a step by step fashion.

## 3. Results and Discussion

We first examined existing personal defense procedures and techniques as reported in major law enforcement officer manuals currently in force in Spain with the above-described medical considerations in mind. The manuals are intended to help officers brush up previously learnt procedures and techniques and also to implement them as physical intervention techniques (PITs) in troublesome situations. Some procedures have evolved into new PITs known as operational tactical procedures (OTPs) [44,54].

### 3.1. Injury-Based Analysis of Common Police PITs

Most of the procedures and techniques described in the manuals examined [45,46,47] originated in martial arts; therefore, they were not specifically developed to control and lock opponents, but rather to knock them out. In addition, the actors used to illustrate some procedures are wearing a martial arts kimono and none of the manuals includes a section on warming up before going into action. This is quite striking since officers rarely have the chance to warm up before an intervention—rather, they usually have to switch from inactive to active within seconds. On medical grounds, they should therefore be trained in alternative procedures and techniques requiring no warm-up in order to prevent unnecessary risks [42,48].

The manuals examined define and describe the risks of causing serious injuries by acting on points of high (red zones in Figure 1) or even medium vulnerability (yellow zones) but recommend a number of basic procedures and techniques based on striking such points—or even vital zones. For example, some advice hitting the mental region with a straight or hooking punch, or hitting the chin or temporal zone with an open hand. Because these blows typically hit the head or face, they can cause external injuries in the scalp or facial skeleton, in addition to severe concussion, hemorrhage and/or encephalic injuries potentially resulting in serious nerve damage or even in immediate or delayed death. Thus, hitting the temple, where the temporal bone—the weakest among cranial bones—lies, can easily break it and cause an encephalic injury. Therefore, targeting striking this zone is unjustified unless no alternative target exists owing to the high associated risk of very serious damage or even death [2,5]. For example, a punch can easily exert a pressure equivalent to 400 kg, which can very easily damage especially sensitive zones such as the face or cranium.

The neck is one other region officers are trained to punch or elbow even though the blow may inflict serious damage to the airways (e.g., a tracheal or laryngeal injury) and/or to vascular (carotid arteries, jugular veins) or nerve structures [9,14]. In addition, a blow on the neck (especially one on the side, where carotid arteries bifurcate) can trigger the carotid sinus and result in sudden fainting—or, as noted earlier, even in cardiac arrest and, ultimately, death [34,46]. However, updated manuals also describe choking techniques that can elicit a response from the carotid sinuses and result in a vagal discharge, thereby easily damaging bony structures such as the cervical spine. The resulting injury can range from cervical sprain to rib fracture and lead to highly severe damage of the spine cord with very serious consequences in some cases. The neck is therefore another vital region that should not be stricken or pressed as far as possible [16,17,18]

Other techniques dealt with in the manuals involve blows on the chest or in the abdomen with a front, side, back or circular kick, a kick stamp or a straight or hooking knee blow. A kick in the abdomen can apply a pressure equivalent to 700 kg—or even more if the typical heavy officer boots are worn—and a knee blow up to 900 kg. Therefore, kicking or kneeing the abdomen has a very high risk of causing a very serious injury such as liver or spleen fracture—and its associated hemorrhage—or damage to the kidneys or the lumbar vertebral spine [29]. In addition, the epigastrium, where the stomach lies, is in the solar plexus, whose striking can trigger a response altering the heart rate and blood pressure to an extent leading to death through hypotension and bradycardia, respectively [31,32]. Updated manuals advise elbowing the solar plexus and describe the applicable technique in detail despite its high associated risk.

Similarly, kicking or kneeing the chest can not only result in bone injury but also in severe lung trauma leading to hemorrhage or contusion—or even to a fatal pneumothorax or haemothorax [22,27]. Further, a blow in the precordial zone, where the heart is, can result in cardiac trauma, which, in addition to contusion and direct injury, may cause serious heart rate disturbances and lead to commotion cordis, a condition potentially leading to death within minutes [23,37]. Based on the foregoing, these techniques should not be applied to the chest or abdomen if their intrinsic risks are to be avoided, and people’s lives and physical integrity preserved.

The manuals also depict throwing techniques aimed at sending opponents to the ground by using appropriate locks or holds to break their balance. Being suddenly thrown to the ground and landing on a hard surface can cause head or spine trauma, and the trauma be worsened by bumping on, for instance, furniture or a curb [49].

In addition, there are also descriptions in the manuals for control techniques, which involve dislocating a wrist, elbow or knee in order to lock the individual to be arrested. These techniques are applied to zones of medium vulnerability (yellow zones in Figure 1). Although the joints acted upon can easily be damaged, the injuries are rarely serious. The manuals, however, fail to recommend application of these techniques acting on yellow zones, and also to emphasize the potential dangers of acting on vulnerable points.

One other procedure worth noting here is that used to arrest people in motion. While the target individual is unaware, the officer walks to him, grabs one of his wrists and uses his other hand to punch the arrestee. Once the individual has been thrown to the ground and placed into ventral decubitus, the officer rests both knees on him to exert pressure on his neck and back in order to lock him. This procedure, by which the officer’s whole weight rests on the arrestee’s, still in ventral decubitus, can also be used for shackling or even frisking. Hitting the face and pressing the arrestee’s neck and face have a high risk of inflicting severe injury or even death, however [2,7]. In fact, pressing the opponent’s neck under one’s knee may result in very serious cervical injuries, impair breathing, hinder blood flow to the brain and, ultimately, death, especially if the arrestee’s resists locking with brusque movements [9,12,14]. Likewise, pressing the arrestee’s back or chest may hinder breathing and lead to suffocation. Very similar injuries can result from the officer’s lying aplomb on the back and neck of the arrestee, who should previously have been placed into ventral decubitus. The potential adverse consequences of this procedure include severe damage or even death if the arrestee’s has to bear the weight of several officers at once [27,28]. This is, thus, a controversial procedure involving serious risks that officers should be aware of in order to ensure that the arrestee’s can breathe properly, and also so that serious neck or spine injuries are avoided.

Finally, some manuals deal with “pressure points”. These are vital points officers can hit if needed but are best not targeted to avoid irreversible damage. As stated in the manuals, officers should know where pressure points fall; however, they should think of them as targets to be avoided rather than sought. One such point is the temple, which should only be hit with an open hand [3]. One other point is the trachea, a blow in which may obstruct or even break it and result in death through suffocation—which is why it should only be hit with the V of the hand [18]. Similarly, striking a carotid sinus can result in a loss of motor coordination and in fainting [13]. As noted earlier, overstimulating a reflex-triggering zone can cause a vagal discharge and lead to death through cardiorespiratory arrest (especially when, as recommended in the manuals, it results from elbowing of the carotid). A further pressure point is the pit of the stomach, a reflex-triggering zone in the solar plexus that contains the epigastrium and hitting of which has an associated risk of death. Similarly fragile are the liver and spleen, which can be easily fractured and lead to death through hemorrhage [28,31]. All these vital points should therefore be avoided because their hitting can easily have a fatal outcome. Based on the numerous manuals and procedures focused on the zones of high vulnerability it can be observed that the rate of serious injuries is pretty high.

### 3.2. The Principles of Congruence, Opportunity and Proportionality

Based on the foregoing, and on the police’s commitment to preserving people’s lives and physical integrity, all police actions should comply with the principles of congruence, opportunity and proportionality. This requires advising law enforcement officers and private security guards to act on non-vulnerable points (viz., points in the upper and lower limbs excluding the hands, wrists, knees and feet if at all possible) when applying the above-described potentially dangerous procedures in order to avoid unwanted serious injuries.

In fact, the upper and lower limbs contain pressure points whose pressing may cause strong enough pain to render an individual motionless by effect of their being richly innervated. These non-vulnerable points include large muscles such as the femoral quadriceps, brachial triceps or glutei, large tendon insertion zones such as triceps or lateral vast tendon insertions (indicated in Figure 1) and others such as the anterior tibial edge, trauma of which can be highly painful even in the absence of injury [43,49]. Similarly efficient and undamaging is acting on large joints such as the shoulder, which is less prone to injury under locking (e.g., when handcuffing, tying or shackling). Trauma in these zones can result in apparent injuries such as contusion, ecchymosis, rupturing of fibrils, tendons or muscles, and dislocation. Most often, however, the damage is not serious relative to highly vulnerable or vital zones (red zones in Figure 1) [40,41]. In summary, opponents should be locked, and arrested if needed, by applying effective techniques to non-vulnerable points. Therefore, law enforcement officers should be instructed in procedures where the use of physical strength is replaced with weight shifts and pain control in acting on especially sensitive zones and breaking the balance of the individual to be subdued.

### 3.3. Physical Intervention Techniques Based on Operational Tactical Procedures

Based on the previous considerations, an OTP comprising a series of physical intervention techniques (PITs) for locking and arresting that target non-vulnerable points has been developed. The proposed PITs use defense mechanisms such as blocking, diverting and grabbing of the upper and lower limbs while avoiding vulnerable and vital zones. In fact, the procedures described in OTP manuals rely on inflicting controlled pain by acting on non-vulnerable points that are especially sensitive by virtue of their rich innervation. Thus, hitting the triceps tendon or the quadriceps muscle can be painful enough for the opponent to be blocked and completely controlled. These procedures avoid serious damage not only of the opponent but also of the officer. In addition, unlike martial arts techniques, they require no warm-up, so they reduce the risk of officers being injured [48].

Based on the foregoing, three different PITs to be applied to green (non-vulnerable) zones (i.e., zones that are much less prone to damage than the red and yellow zones of Figure 1) are proposed to avoid serious injuries or even death while ensuring that an opponent is arrested without damage to the officer. Although these PITs can be replaced with others if needed in specific situations, they cater for most types of troublesome situations officers can face in an urban scenario.

#### 3.3.1. Technique 1. Response to a Hooked Punch

**Figure 2:**
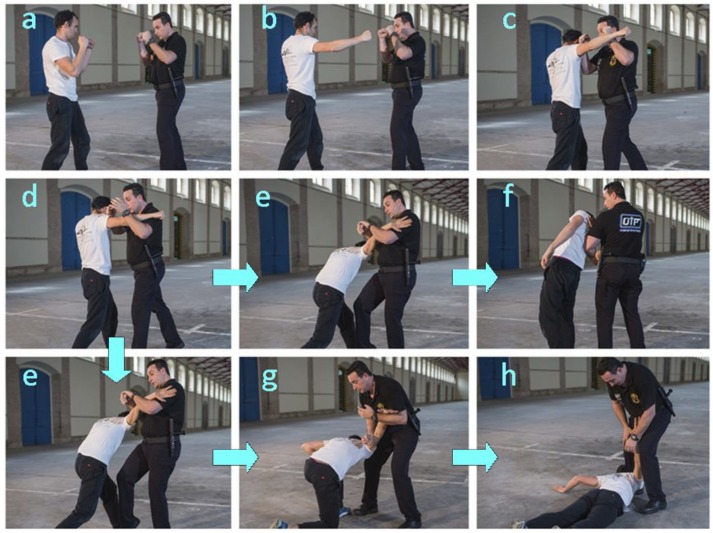
Steps of physical intervention techniques (PIT) 1 (response to a hooking punch). (**a**) The officer faces the opponent. (**b**) The opponent charges against the officer. (**c**) The officer with his forearms blocks the opponent’s forward arm. (**d**) The officer releases the arm he previously used to block the opponent’s arm. (**e**) The officer’s previously released arm is used to grab the opponent break his balance with a knee blow on the opponent’s backward leg. After this picture the arrows indicate the flow of motion between the officer and opponent. The latter can be subdued in two different ways (as are pictured in f or g-h). (**f**) While standing, the officer controls the opponent by using his forearm to exert strong pressure on the opponent’s elbow. (**g**) The officer controls the opponent with the same forearm in order to lower it and keep it bent. (**h**) The officer continues to exert further pressure with the forearm to take the opponent to the ground.

As illustrated by the image sequence of Figure 2, the technique involves the following steps:

– Step 1. The officer faces the opponent in the guard stance (Figure 2a).
– Step 2. The officer approaches the opponent and blocks his forward arm with his forearms (Figure 2c) in response to the opponent’s charge (Figure 2b).
– Step 3. The officer releases the arm he previously used to block the opponent’s arm (Figure 2d).
– Step 4. The officer’s previously released arm is used to grab the opponent break his balance with the help of a knee blow on the opponent’s backward leg, in particular on the frontal area of the thigh (Figure 2e).
– Step 5. The opponent’s is subdued in either of two different ways depending on his aggressiveness:
  - 5A. While standing (Figure 2f). The officer controls the opponent by using his forearm to exert strong pressure on the opponent’s elbow and also to prop himself up in order to lower the elbow to his own hip level.
  - 5B. On the ground (in one or two steps). The officer continues to control the opponent with the same forearm in order to lower it and keep it bent, still under control, at the officer’s knee level (Figure 2g) while exerting further pressure with the forearm to take the opponent to the ground (Figure 2h).

#### 3.3.2. Technique 2. Response to a Knife Blow

**Figure 3:**
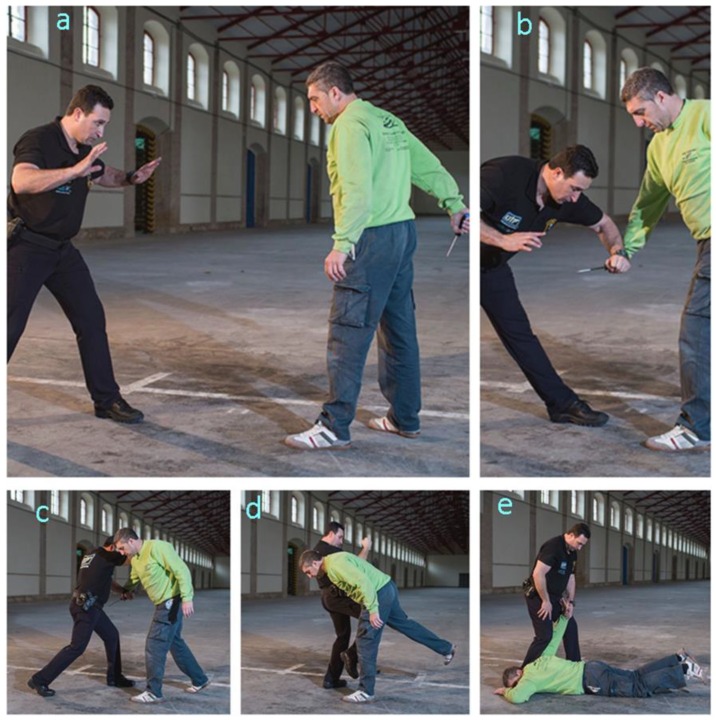
Steps of PIT 2 (response to a knife blow). (**a**) The officer faces the opponent holding a knife. (**b**) The officer uses one forearm to block the arm holding the knife. (**c**) The officer uses his free forearm to block the opponent’s free elbow. (**d**) Once the opponent is controlled, his balance is broken by kneeing on the backward leg. (**e**) Subduing the opponent on the ground.

The sequence of Figure 3 illustrates the procedure, which comprises five steps, namely:

– Step 1. The officer adopts the guard stance while facing the opponent, who is holding a knife (Figure 3a).
– Step 2. The officer uses one forearm to block the arm holding the knife (Figure 3b).
– Step 3. The officer approaches the opponent and uses his free forearm to block the opponent’s free elbow (Figure 3c).
– Step 4. Once the opponent is controlled, his balance is broken by kneeing on the backward leg (Figure 3d).
– Step 5. Subduing the opponent on the ground. The officer controls the opponent by using his forearm to exert strong pressure on the opponent’s elbow while propping himself up in order to lower the elbow to his own hip level. Then, the officer continues to use his forearm in order to further lower the opponent until he reaches the ground (Figure 3e).

#### 3.3.3. Technique 3. Response to a Blunt Object Blow

**Figure 4:**
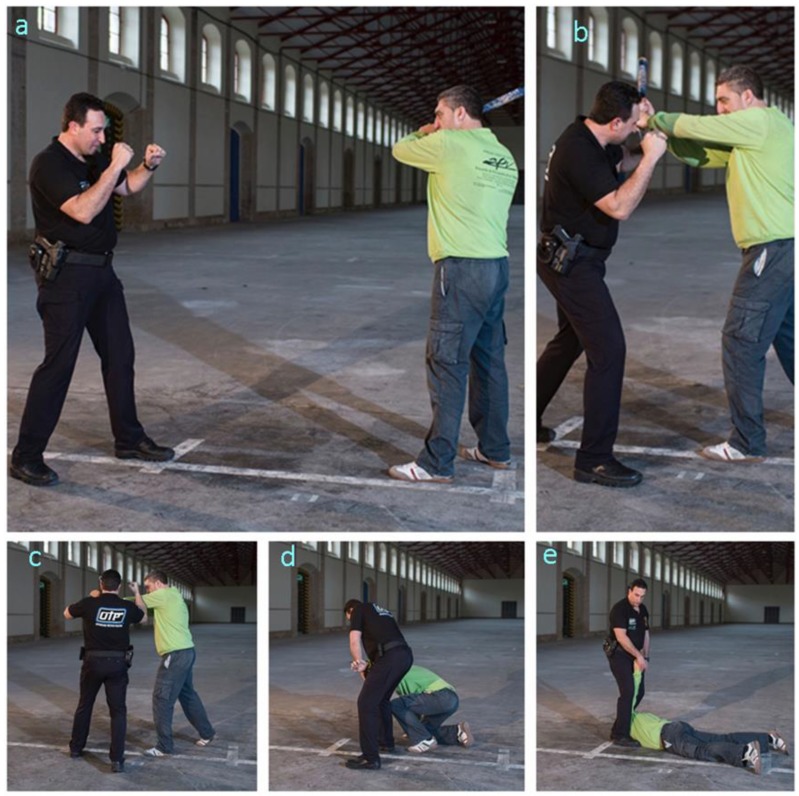
Steps of PIT 3 (response to a blunt object blow). (**a**) The officer faces the opponent holding a weapon. (**b**) The officer moves his left leg forward and uses his left forearm to block the opponent’s blow. (**c**) While grabbing the opponent, the officer uses his right forearm to hit the opponent’s elbow. (**d**) The officer uses his right forearm to exert pressure on the opponent’s triceps. (**e**) The opponent is subdued on the ground similarly as with Techniques 2 and 3.

As illustrated in the image sequence of Figure 4, the procedure involves the following steps:

– Step 1. The officer adopts the guard stance while facing the opponent, who is holding a blunt object as a weapon (Figure 4a).
– Step 2. The officer approaches the opponent from a side, moves his left leg forward and uses his left forearm to block the opponent’s blow (Figure 4b).
– Step 3. While grabbing the opponent with his left hand, the officer uses his right forearm to hit the opponent’s elbow (Figure 4c).
– Step 4. The officer uses his right forearm to exert pressure on the opponent’s triceps in order to gradually drive him to the ground (Figure 4d).
– Step 5. The opponent is subdued on the ground similarly as with Techniques 2 and 3 (Figure 4e).

Officers applying Technique 2 or 3 should wear a padded sleeve for increased safety [Patent 1; ES2615602 (B2)] [55].

The proposed PIT displayed in Figure 1, Figure 2 and Figure 3, avoid the high vulnerability red areas, leading a reduction of the rate of causing serious injuries. All of the proposed techniques are the result of many years of work and were successfully tested in real situations [54]. To do so, police officers from different countries of the world were trained. The effectiveness of the proposed PIT lies not only in preventing injuries to citizens but also in preventing injuries in the police themselves.

## 4. Conclusions

Some training manuals for law enforcement officers and private security agents describe physical intervention techniques (PITs) for use in troublesome situations that carry a high risk of injuring the target individuals. The damaging potential of such PITs was assessed in terms of anatomical region and most were found to have a high risk of severe damage. The alternative PITs proposed here, which rely on operational tactical procedures, improve in legal and forensic medical terms on existing choices and dramatically reduce the risk of injuring arrestees as a result of acting on zones of minimal vulnerability in order to preserve people’s safety and physical integrity.

## 5. Patents

Vera-Jiménez, J.C.; University of Cadiz; Versatile protector suitable for carrying defenses for police use and other accessories, and for the use of defensive blocking techniques. Methods of employment. ES2615602 (B2). 2018-02-01.

## References

[1] Mckee A.C., Cantu R.C., Nowinski C.J., Hedley-Whyte E.T., Gavett B.E., Budson A.E., Santini V.E., Lee H.S., Kubilus C.A., Sern R.A. (2009). Chronic traumatic encephalopathy in athletes: Progressive tauopathy after repetitive head injury. J. Neuropathol. Exp. Neurol..

[2] Langlois J.A., Rutland-Brown W., Wald M.M. (2006). The epidemiology and impact of traumatic brain injury: A brief overview. J. Head Trauma Rehab..

[3] Thurman D.J., Branche C.M., Sniezek J.E. (1998). The epidemiology of sports-related traumatic brain injuries in the United States: Recent developments. J. Head Trauma Rehab..

[4] Salehi A., Zhang J.H., Obenaus A. (2017). Response of the cerebral vasculature following traumatic brain injury. J. Cereb. Blood Flow Metab..

[5] Logsdon A.F., Lucke-Wold B.P., Turner R.C., Huber J.D., Rosen C.L., Simpkins J.W. (2015). Role of microvascular disruption in brain damage from traumatic brain injury. Compr. Physiol..

[6] Saboe L.A., Reid D.C., Davis L.A., Warren S.A., Grace M.G. (1991). Spine trauma and associated injuries. J. Trauma Inj. Infect. Crit. Care.

[7] Pickett W., Simpson K., Walker J., Brison R.J. (2003). Traumatic spinal cord injury in Ontario, Canada. J. Trauma.

[8] Furlan J.C., Sakakibara B.M., Miller W.C., Krassioukov A.V. (2013). Global incidence and prevalence of traumatic spinal cord injury. Can. J. Neurol. Sci..

[9] Fakhry S.M., Jaques P.F., Proctor H.J. (1988). Cervical vessel injury after blunt trauma. J. Vasc. Surg..

[10] Phillips A.A., Krassioukov A.V., Ainslie P.N., Warburton D.E.R. (2012). Baroreflex function after spinal cord injury. J. Neurotraum..

[11] O’Kane J.W. (2001). Syncope following neck trauma in a football player. Phys. Sportsmed..

[12] Kochhar T., Back D.L., Mann B., Skinner J. (2005). Risk of cervical injuries in mixed martial arts. Br. J. Sports Med..

[13] Morley C.A., Sutton R. (1984). Carotid sinus syncope. Int. J. Cardiol..

[14] Conway D., Urquhart C.S. (2017). Airway trauma. Anaesth. Intensive Care.

[15] Corneille M.G., Stewart R.M., Cohn S.M. (2008). Upper airway injury and its management. Semin. Thorac. Cardiovasc. Surg..

[16] Demetriades D., Salim A., Brown C., Martin M., Rhee P. (2007). Neck injuries. Curr. Probl. Surg..

[17] Erdogan B., Erdogan M.O., Colak S., Kibici O., Bozan K., Alper B. (2015). An isolated hyoid bone fracture caused by blunt trauma to the neck. J. Pak. Med. Assoc..

[18] Jehng Y.M., Lee F.T.T., Pai Y.C., Choi W.M. (2012). Hyoid bone fracture caused by blunt neck trauma. J. Acute Med..

[19] Levine E., Taub P.J. (2006). Hyoid bone fractures. Mt. Sinai J. Med..

[20] Moya A., Sutton R., Ammirati F., Blanc J.J., Brignole M., Dahm J.B., Deharo J.C., Gajek J., Gjesdal K., Krahn A. (2009). Guidelines for the diagnosis and management of syncope (version 2009). Eur. Heart J..

[21] Estañol B., Porras Betancourt M., Sánchez Torres G., Martínez Memije R., Infante O., Sentíes Madrid H. (2009). Neural control of the peripheral circulation and blood pressure. Arch. Cardiol. Mex..

[22] Wang N.D., Stevens M.H., Doty D.B., Hammond E.H. (2003). Blunt chest trauma: An experimental model for heart and lung contusion. J. Trauma.

[23] Link M.S. (2012). Commotio cordis: Ventricular fibrillation triggered by chest impact-induced abnormalities in repolarization. Circ. Arrhythm. Electrophysiol..

[24] Link M.S., Wang P.J., Maron B.J., Estes N.A. (1999). What is commotio cordis?. Cardiol. Rev..

[25] Shorr R.M., Crittenden M., Indeck M., Hartunian S.L., Rodríguez A. (1987). Blunt thoracic trauma. Analysis of 515 patients. Ann. Surg..

[26] Liman S.T., Kuzucu A., Tastepe A.I., Ulasan G.N., Topcu S. (2003). Chest injury due to blunt trauma. Eur. J. Cardiothorac. Surg..

[27] Freixinet Gilart J., Elena Ramírez Gil M., Gallardo Varela G., Moreno Casado P. (2011). Chest trauma [Traumatismos torácicos]. Arch Bronconeumol..

[28] Lin F.C.F., Li R.Y., Tung Y.W., Jeng K.C., Tsai S.C.S. (2016). Morbidity; mortality; associated injuries; and management of traumatic rib fractures. J. Chin. Med. Assoc..

[29] Prachalias A.A., Kontis E. (2014). Isolated abdominal trauma: Diagnosis and clinical management considerations. Curr. Opin. Crit. Care.

[30] Jordan G.L., Beall A.C. (1971). Diagnosis and management of abdominal trauma. Curr. Probl. Surg..

[31] Mercadante S., Nicosia F. (1998). Celiac plexus block: A reappraisal. Regional Anesth..

[32] De Froidmont S., Lobrinus J.A., Michaud K., Palmiere C., Augsburger M.P., Mangin P., Grabherr S. (2015). Cardioinhibitory reflex due to a karate kick. Am. J. Forensic Med. Pathol..

[33] Schrag B., Vaucher P., Bollmann M.D., Mangin P. (2011). Death caused by cardioinhibitory reflex cardiac arrest—A systematic review of cases. Forensic Sci. Int..

[34] Hess D.S., Hanlon T., Scheinman M., Budge R., Desai J. (1982). Termination of ventricular tachycardia by carotid sinus massage. Circulation.

[35] Schrag B., Mangin P., Vaucher P., Bollmann M.D. (2012). Death caused by cardioinhibitory reflex: What experts believe. Am. J. Forensic Med. Pathol..

[36] Gaggioli G., Brignole M., Menozzi C., Bottoni N., Gianfranchi L., Oddone D., Lolli G. (1995). Reappraisal of the vasodepressor reflex in carotid sinus syndrome. Am. J. Cardiol..

[37] Lucena J.S., Rico A., Salguero M., Blanco M., Vázquez R. (2008). Commotio cordis as a result of a fight: Report of a case considered to be imprudent homicide. Forensic Sci. Int..

[38] Bergqvist D., Hedelin H., Karlsson G., Lindblad B., Matzsch T. (1982). Abdominal injury from sporting activities. Br. J. Sports Med..

[39] Kazemi M., Shearer H., Young S.C. (2005). Pre-competition habits and injuries in Taekwondo athletes. BMC Musculoskelet. Disord..

[40] Kazemi M., Pieter W. (2004). Injuries at a Canadian National Taekwondo Championships: A prospective study. BMC Musculoskelet. Disord..

[41] Mithofer K., Lhowe D.W., Altman G.T. (2002). Delayed presentation of acute compartment syndrome after contusion of the thigh. J. Orthop. Trauma..

[42] Čierna D., Barrientos M., Agrasar C., Arriaza R. (2018). Epidemiology of injuries in juniors participating in top-level karate competition: A prospective cohort study. Br. J. Sports Med..

[43] Hübscher M., Zech A., Pfeifer K., Hänsel F., Vogt L., Banzer W. (2010). Neuromuscular training for sports injury prevention: A systematic review. Med. Sci. Sports Exerc..

[44] Vera-Jiménez J.C. Manual Intervención Policial y Prevención de Riesgos—OTP. http://d-nb.info/1121789234.

[45] Benito J.M. Manual Básico de Procedimientos y Defensa Personal Policial I and Manual Básico de Procedimientos y Defensa Personal Policial ii. Cuerpo Nacional de Policía. https://es.scribd.com/document/74168214/Manual-Defensa-Personal-Policial2.

[46] Manual Defensa Personal Guardia Civil para la Academia de Guardias y Suboficiales de la Guardia Civil (2010). Jefatura de Enseñanza.

[47] Plan Anual de Formación de la Escuela de Seguridad Pública de Andalucía Para el Curso Académico 2015. http://www.juntadeandalucia.es/organismos/justiciaeinterior/areas/policia/espa/paginas/plan-formacion-espa.html.

[48] Buschbacher R.M. (1999). Martial arts. Phys. Med. Rehabil. Clin. N. Am..

[49] Pieter W. (2005). Martial arts injuries. Med. Sport Sci..

[50] Hoover E.J. (1951). Defensive tactics. A Handbook for Law Enforcement Officers.

[51] Levine D., Whitman J. (2016). Complete Krav Maga.

[52] MIEMIO—Ministerio del Interior de España Manual de Intervención Operativa (2014). Ministerio del Interior.

[53] Vera Jiménez J.C., Fernández F., Ayuso J., Lorente J.A. (2020). Evaluation of the police operational tactical procedures for reducing officer injuries resulting from physical interventions in problematic arrests. The case of the Municipal Police of Cádiz (Spain). Int. J. Occup. Med. Environ. Health.

[54] Vera-Jiménez J.C., Ferreiro-González M., Barbero G.F., Álvarez J.Á., Fernández-Zacarías F., Ayuso J. (2019). OTP-PRL: An app for occupational risk prevention in policing activities. BMC Public Health.

[55] Patents. https://patents.google.com/patent/ES2615602B2/es?oq=ES2615602B2.

